# The long non-coding RNA HNF1A-AS1 regulates proliferation and metastasis in lung adenocarcinoma

**DOI:** 10.18632/oncotarget.3247

**Published:** 2015-03-26

**Authors:** Ying Wu, Hongbing Liu, Xuefei Shi, Yanwen Yao, Wen Yang, Yong Song

**Affiliations:** ^1^ Department of Respiratory Medicine, Jinling Hospital, Nanjing University School of Medicine, Nanjing, China

**Keywords:** lncRNAs, HNF1A-AS1, proliferation, metastasis, lung adenocarcinoma

## Abstract

Long noncoding RNAs (lncRNAs) have emerged as key regulators of tumor development and progression. The lncRNA HNF1A-antisense 1 (HNF1A-AS1) is a 2455-bp transcript on chromosome 12 with a potential oncogenic role in esophageal adenocarcinoma. Nevertheless, current understanding of the involvement of HNF1A-AS1 in lung adenocarcinoma tumorigenesis remains limited. In this study, we analyzed the roles of HNF1A-AS1 in 40 lung adenocarcinoma tissues and five lung cancer cell lines. Our results showed that HNF1A-AS1 was significantly up-regulated in lung adenocarcinoma tissues compared with corresponding non-tumor tissues, and its expression level was significantly correlated with TNM stage, tumor size, and lymph node metastasis. The UCSC Cancer Genomics Browser's Kaplan-Meier plot suggested that patients in the high HNF1A-AS1 expression subgroup experienced worse overall survival compared to the low expression subgroup. Moreover, HNF1A-AS1 was determined to promote tumor proliferation and metastasis, both *in vitro* and *in vivo*, by regulating cyclin D1, E-cadherin, N-cadherin and β-catenin expression. In addition, the binding of HNF1A-AS1 to DNMT1 may explain its regulation of E-cadherin. In conclusions, we demonstrated that increased HNF1A-AS1 expression could regulate cell proliferation and metastasis and identified it as a poor prognostic biomarker in lung adenocarcinoma.

## INTRODUCTION

Lung cancer is the most common cause of global cancer-related mortality with an approximate 5-year survival rate of 16.6%, and lung adenocarcinoma is its most common histological type (http://www.cancer.gov/cancertopics/pdq/treatment/non-small-cell-lung/healthprofessional/page2) (http://seer.cancer.gov/publications/csr.html) [1, 2]. A critical problem in the progression of lung adenocarcinoma is the metastasis of cancer cells, which determines patients’ prognosis. Therefore, understanding the mechanisms and molecular pathways underlying lung adenocarcinoma progression and metastasis is crucial for improving the therapy and overall prognosis of this disease.

Cancer cell invasion and metastasis are complex processes involving many factors, both cellular factors and extrinsic factors related to the microenvironment. One of the most critical molecular mechanisms mediating the metastatic cascade is the epithelial-mesenchymal transition (EMT) in cancer cells, which is also a primary step in the induction of tumor cell invasion and metastasis *in situ* [3]. Similarly to the EMT during embryonic development, the EMT in cancer cells enables them to overcome cell-cell adhesion and increases cell motility, endowing epithelial cancer cells with a mesenchymal phenotype [4]. Furthermore, the EMT is characterized by important hallmark changes including decreased E-cadherin expression and increased expression of non-epithelial cadherins such as N-cadherin. Importantly, the loss of E-cadherin expression is a rate-limiting step in the progression of well-differentiated adenoma to invasive carcinoma [5]. To date, most studies investigating how the EMT is regulated in cancer have focused on specific protein-encoding genes or some signaling pathways, for instance epidermal growth factor (EGF), transforming growth factor (TGF)-β, and fibroblast growth factor (FGF) [6]. However, recent evidence has accumulated indicating that long non-coding RNAs (LncRNA) may be involved in non-small cell lung cancer (NSCLC) pathogenesis, providing a new understanding of the molecular mechanisms underlying lung adenocarcinoma metastasis [7, 8].

The development of microarrays and high-throughput sequencing revealed that > 90% of the total mammalian genome can be transcribed into non-coding RNAs [9]. Among these non-coding RNAs are lncRNAs, which are more than 200 nt in length with little protein-coding potential [10]. Although few lncRNAs have been described in detail, many lncRNAs have been shown to perform various tasks including cellular development, modulation of apoptosis and metastasis, and parental imprinting [11–13]. Emerging evidence suggests that the dysregulation of lncRNAs is linked with the development and metastasis of various types of cancers including lung cancer [14–17], making a complete understanding of their biological functions in EMT important for understanding the molecular biology of lung adenocarcinoma metastasis and progression.

The long non-coding RNA HNF1A-AS1 (HNF1A antisense RNA 1, C12orf27) located on chromosome 12 is transcribed as a 2.455 kb lncRNA in the opposite direction of HNF1A gene transcription [18]. (http://www.ncbi.nlm.nih.gov/gene/?term=HNF1A-AS1) Recently, a next-generation sequencing analysis identified the HNF1A-AS1 gene as a non-coding oncogene involved in the tumorigenesis of esophageal adenocarcinoma [19]. Yang et al. have shown that higher levels of lncRNA HNF1A-AS1 expression were observed in esophageal adenocarcinoma, and they described the effects of this lncRNA on cell proliferation, cell cycle regulation, migration and invasion. They also proved that lncRNA H19 may represent a downstream effector of HNF1A-AS1. These results indicated that the dysregulation of HNF1A-AS1 could participate in esophageal adenocarcinoma. However, the functional role and underlying mechanism of HNF1A-AS1 in lung adenocarcinoma remain unclear.

In the present study, we showed that HNF1A-AS1 is up-regulated in lung adenocarcinoma tissues compared with corresponding non-tumor tissues and that its expression level is significantly correlated with TNM stage, tumor size, and lymph node metastasis. Moreover, HNF1A-AS1 could regulate cell growth and metastasis both *in vitro* and *in vivo* by regulating cyclin D1, E-cadherin, N-cadherin and β-catenin expression. In addition, we demonstrated that HNF1A-AS1 could bind to DNA methyltransferase (cytosine-5) 1 (DNMT1), and this binding could explain its regulation of E-cadherin. Our results suggest that increased HNF1A-AS1 expression may play an important role in lung adenocarcinoma carcinogenesis and metastasis.

## RESULTS

### HNF1A-AS1 is up-regulated in lung adenocarcinoma tissues and correlates with poor prognosis

**Figure 1:**
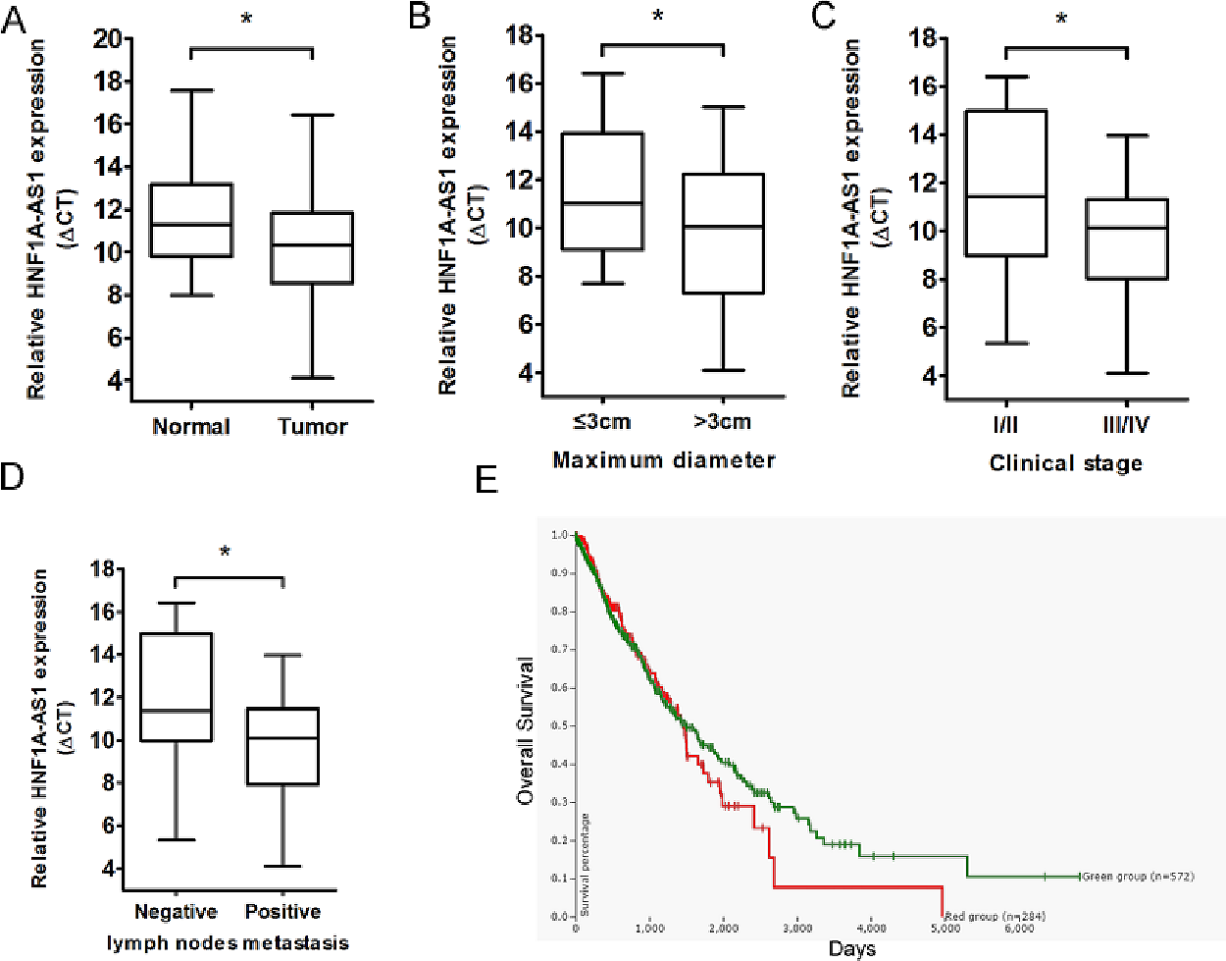
Relative HNF1A-AS1 expression in lung adenocarcinoma tissues and its clinical significance **(A)** Relative expression of HNF1A-AS1 expression in lung adenocarcinoma tissues (*n* = 40) and in paired adjacent normal tissues (*n* = 40). HNF1A-AS1 expression was examined by qPCR and normalized to GAPDH expression. (shown as ΔCT) **(B–D)** HNF1A-AS1 expression was significantly higher in patients with big tumor size, advanced clinical stage and lymph nodes metastasis. **(E)** The Kaplan-Meier plot in UCSC Cancer Genomics Browser indicated that HNF1A-AS1 high expression (red line) has a worse overall survival compared to the low expression subgroup (green line). See https://genome-cancer.ucsc.edu/proj/site/hgHeatmap/#? bookmark=3c7d51368bb7caa36d17e4c957103893. *p < 0.05.

To validate whether HNF1A-AS1 was differentially expressed in lung adenocarcinoma tissues, a total of 40 paired clinical lung adenocarcinoma tissues and adjacent normal counterparts were analyzed for HNF1A-AS1 expression using qRT-PCR. HNF1A-AS1 expression was significantly over-regulated in cancerous tissues (*p* = 0.03; Figure 1A). In addition, to evaluate the clinical significance of HNF1A-AS1, we assessed the correlation of its expression with clinicopathological parameters (i.e., stage, maximum diameter and lymph node metastasis). As shown in Figure 1B, 1C, 1D and Table 1, HNF1A-AS1 expression levels in lung adenocarcinoma were significantly associated with tumor size (*p* = 0.022), TNM stage (*p* = 0.046), and lymph node metastasis (*p* = 0.011). However, HNF1A-AS1 expression was not correlated with other clinical characteristics such as differentiation (*p* = 0.354), gender (*p* = 0.722) or age (*p* = 0.505) in lung adenocarcinoma (Table 1).

**Table 1:** Correlation between HNF1A-AS1 expression and clinicopathological parameters of lung adenocarcinoma patients

Characteristics	N of cases	Relative HNF1A-AS1 expression		
		Low	High	*P*-value^a^
**Age(years)**				0.505
≤ 65	24	8	16	
> 65	16	7	9	
**Gender**				0.722
male	28	10	18	
female	12	5	7	
**Differentiation**				0.354
well, moderate	25	8	17	
poor	15	7	8	
**Tumor size (maximum diametercm)**				0.022^*^
≤ 3cm	20	11	9	
> 3cm	20	4	16	
**Smoking History**				0.804
smokers	23	9	14	
never Smokers	17	6	11	
**Lymph node metastasis**				0.011^*^
positive	21	4	17	
negative	19	11	8	
**TMN stage**				0.046^*^
I	16	9	7	
II/III/IV	24	6	18	

a Chi-square test.

* *P* < 0.05

Using the UCSC Cancer Genomics Browser's genomic signature function, we defined gene expression signatures of C12orf27, aliases of HNF1A-AS1 [18]. The browser's Kaplan-Meier plot suggests that the patients in the high HNF1A-AS1 expression subgroup (red line) have worse overall survival compared to the low expression subgroup (green line). See https://genome-cancer.ucsc.edu/proj/site/hgHeatmap/#?bookmark=3c7d51368bb7caa36d17e4c957103893 (Figure 1E).

These data demonstrate that the upregulation of HNF1A-AS1 may play important roles in lung adenocarcinoma development and progression.

### HNF1A-AS1 expression in NSCLC cells

**Figure 2:**
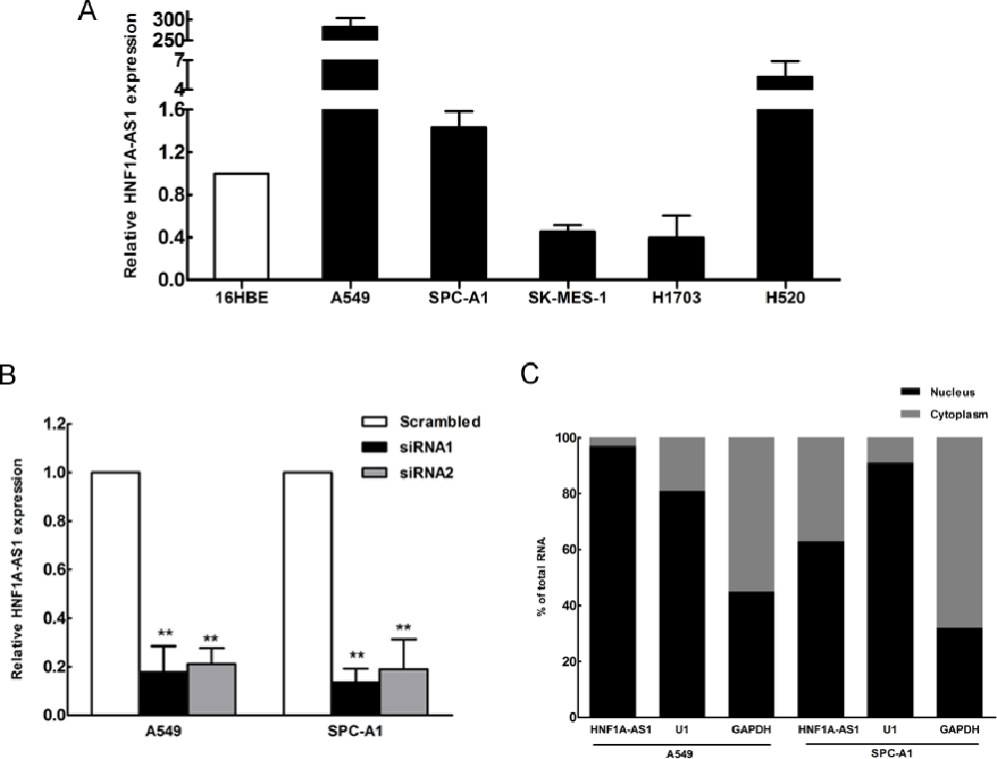
HNF1A-AS1 expression in non-small cell lung cancer (NSCLC) cells **(A)** qRT-PCR results demonstrating HNF1A-AS1 expression in NSCLC cell lines (A549, SPC-A1, SK-MES-1, H1703, H520) compared to human bronchial epithelial cells (16HBE). **(B)** HNF1A-AS1 expression, measured by qRT-PCR, following the treatment of A549 and SPC-A1 cells with scramble or with si-RNA HNF1A-AS1. **(C)** HNF1A-AS1 nuclear localization, as identified using qRT-PCR in fractionated A549 and SPC-A1 cells. GAPDH was used as a cytosol marker and U1 was used as a nucleus marker. *p < 0.05, **p < 0.01.

To explore the role of HNF1A-AS1 in the development of lung cancer, we next performed qRT-PCR analysis to assess HNF1A-AS1 expression in NSCLC cell lines, including both adenocarcinoma and squamous carcinoma subtypes. Compared with that in 16HBE cells, HNF1A-AS1 expression was at a comparatively high level in two lung adenocarcinoma cell lines, including A549, SPC-A1 and the squamous cell carcinoma line H520. In contrast, the relative expression levels of HNF1A-AS1 were decreased in SK-MES-1 and H1703 cell (Figure 2A). To down-regulate endogenous HNF1A-AS1 expression in lung adenocarcinoma cells, small interfering RNAs (siRNAs) were transfected into A549 and SPC-A1 cells. At 48 h post-transfection, HNF1A-AS1 expression was knocked down by approximately 80% in A549 and SPC-A1 cells by si-HNF1A-AS1 transfection when compared with the scrambled siRNA (Figure 2B). We measured HNF1A-AS1 expression in nuclear and cytosolic fractions from A549 and SPC-A1 cells by qRT-PCR. The differential enrichments of GAPDH and U1 RNA were used as fractionation indicators. We observed a considerable increase in HNF1A-AS1 expression in the nucleus versus the cytosol (Figure 2C), thus suggesting that HNF1A-AS1 was mainly localized in the nucleus and maybe played a major regulatory function at the transcriptional level.

### The effect of HNF1A-AS1 on cell proliferation in lung adenocarcinoma cell lines

**Figure 3:**
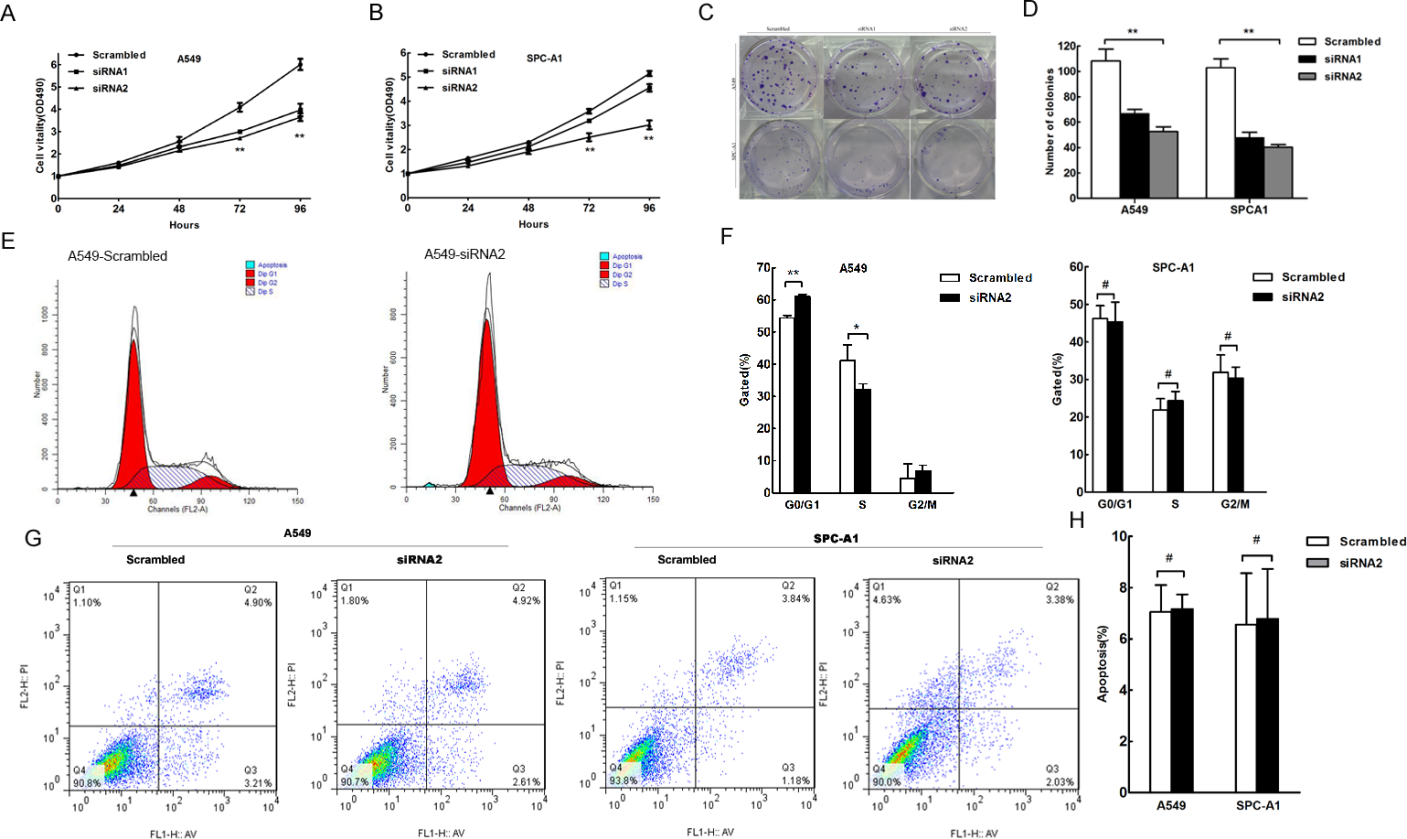
The effect of HNF1A-AS1 on lung adenocarcinoma cell proliferation, cell cycle and apoptosis *in vitro* A549 and SPC-A1 cells were transfected with scramble or si-RNA HNF1A-AS1, respectively. **(A)** and **(B)** MTT assay was performed to determine the proliferation of si-HNF1A-AS1-transfected A549 and SPC-A1 cells. **(C)** and **(D)** A colony-forming growth assay was performed to determine the proliferation of scramble or si-RNA HNF1A-AS1-transfected A549 and SPC-A1 cells. The colonies were counted and captured. **(E)** and **(F)** Cell-cycle analysis was performed 48 h following the treatment A549 and SPC-A1 cells with scramble or si-RNA HNF1A-AS1. The DNA content was quantified by flow cytometric analysis. The data represent the mean ± SD from three independent experiments. **(G)** and **(H)** Apoptosis was determined by flow cytometry. UL, necrotic cells; UR, terminal apoptotic cells; LR, early apoptotic cells. *p < 0.05, **p < 0.01.

As human lncRNAs are involved in a variety of biological processes, we explored the impact of HNF1A-AS1 knock-down in the lung adenocarcinoma cell lines. Compared to the scrambled siRNA-transfected cells, HNF1A-AS1 knockdown resulted in a significant decrease in A549 and SPC-A1 cell viability as monitored by a MTT assay (Figure 3A and 3B). Colony formation assay results also revealed that HNF1A-AS1 down-regulation greatly attenuated clonogenic survival (Figure 3C and 3D). Furthermore, the growth inhibition of A549 cells was accompanied by a corresponding increase in the proportion of cells in G1 and a decrease in the proportion of cells in the S phase. However, siRNA-mediated knockdown of HNF1A-AS1 in SPC-A1 did not promote G1 arrest (Figure 3E and 3F). Additionally, flow cytometry analysis of A549 and SPC-A1 cells showed that downregulation of HNF1A-AS1 expression did not induce apoptosis in comparison with the control cells (Figure 3G and 3H).

Taken together, HNF1A-AS1 over-expression promoted cell proliferation in the lung adenocarcinoma cell lines, partially accompanied by G1 arrest.

### HNF1A-AS1 knock-down suppressed lung adenocarcinoma tumor growth *in vivo*

**Figure 4:**
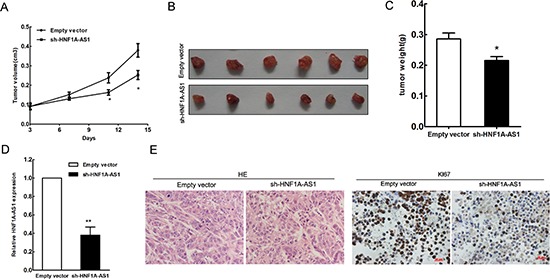
HNF1A-AS1 inhibits tumor growth *in vivo* **(A)** Tumor growth curve. A549 cells were transfected with empty vector or shRNA HNF1A-AS1, and then injected into nude mice (*n* = 7), respectively. Tumor growth was calculated from day 3 after injecting tumor cells. The error bars indicate the standard deviation (SD). **(B)** Total number of tumors after removal from the mice. **(C)** Tumor weight were represented as means mean ± S.D when the tumors were harvested. **(D)** qRT-PCR analyses indicated that HNF1A-AS1 expression is significantly decreased *in vivo*. **(E)** Representative images (×200) of HE staining of the tumor. Immunohistochemistry showed HNF1A-AS1 knockdown decreased the proliferation index Ki67.

To further explore whether the inhibition of HNF1A-AS1 expression can affect tumorigenesis *in vivo*, A549 cells stably transfected with sh-HNF1A-AS1 or empty vectors were inoculated into male nude mice. Fourteen days after injection, HNF1A-AS1 knock-down dramatically inhibited tumor growth compared to the control group, as demonstrated by substantially reduced tumor size and weight (Figure 4A–4C). Moreover, tumor cells was determined by hematoxylin and eosin (HE) staining, and immunohistochemical staining of resected tumor tissues found that tumors formed from sh-HNF1A-AS1-transfected A549 cells exhibited reduced positivity for Ki67 compared with those formed from control cells (Figure 4E). The down-regulation of HNF1A-AS1 in tumors after sh-HNF1A-AS1 transfection was confirmed by qRT-PCR analysis (Figure 4D). Thus, HNF1A-AS1 down-regulation reduces the growth of established lung adenocarcinoma xenografts.

### HNF1A-AS1 knockdown suppresses lung adenocarcinoma metastasis *in vitro* and *in vivo*

**Figure 5:**
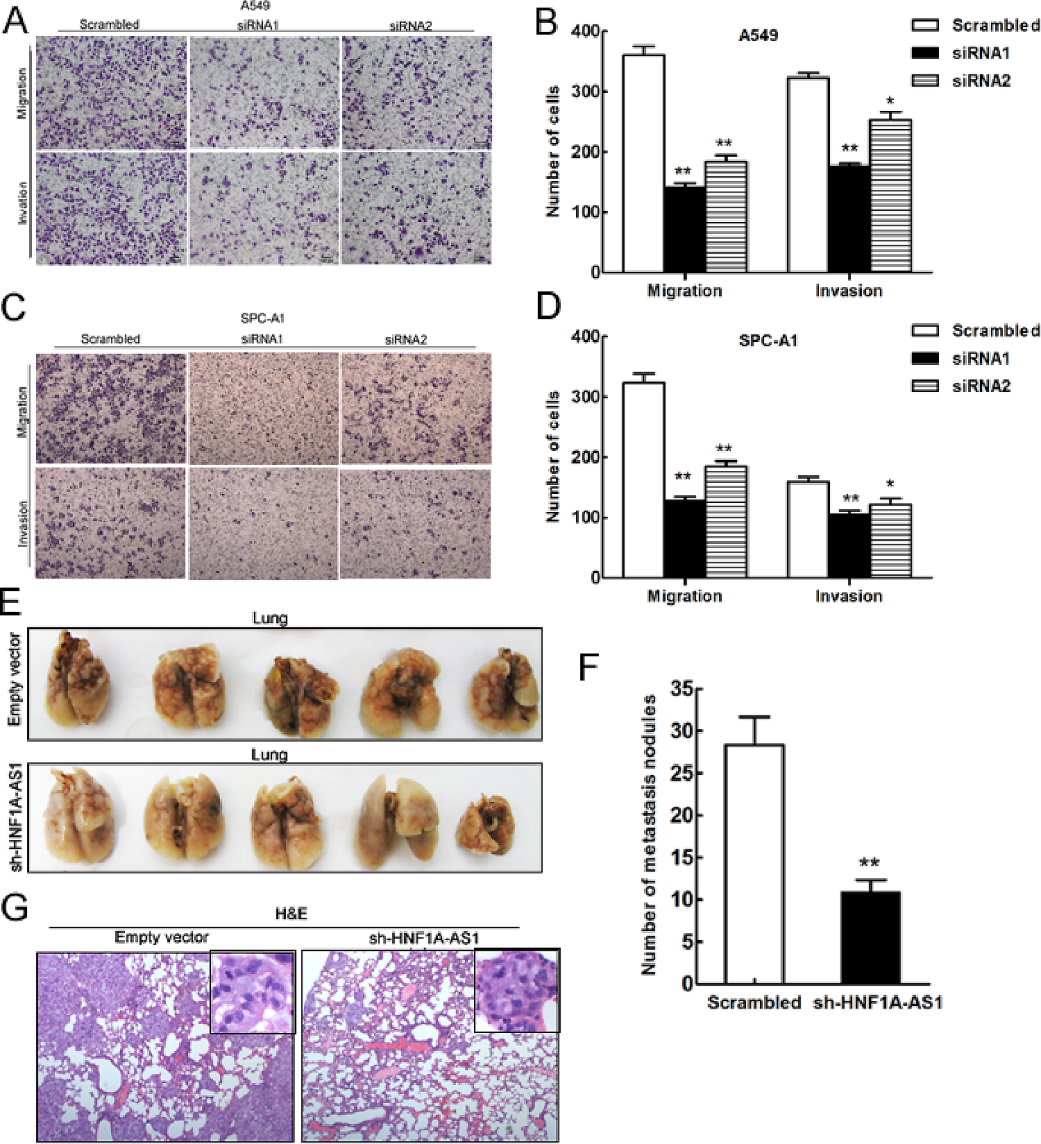
Effects of HNF1A-AS1 downexpression on tumor metastasis *in vitro* and *in vivo* **(A, B, C)** and **(D)** Transwell assays were performed to determine the migratory and invasive abilities of si-HNF1A-AS1-transfected A549 and SPC-A1 cells. **(E)** and **(F)** Analysis of an experimental metastasis animal model was performed by injecting HNF1A-AS1 knockdown A549 cells into nude mice. Lungs from mice in each experimental group, with the numbers of tumor nodules on lung surfaces were shown. **(G)** Visualization of the HE-stained lung sections. **p < 0.01.

To determine whether the inhibition of HNF1A-AS1 expression can promote NSCLC migration and invasion, we evaluated cancer cell invasion through Matrigel and migration through Transwells. Decreased HNF1A-AS1 expression impeded A549 cell migration by 61% and 49% when knocked down by siRNA1 and siRNA2, respectively (Figure 5A and 5B), and by 61% and 43% in SPC-A1 (Figure 5C and 5D). Similarly, A549 cell invasion was also reduced by 45% and 21% by siRNA1 and siRNA2, respectively (Figure 5A and 5B). Down-regulation of HNF1A-AS1 expression also impaired the invasion of SPC-A1 by 34% and 23% by siRNA1 and siRNA2 (Figure 5C and 5D). These findings support the conclusion that HNF1A-AS1 exerts a critical effect on the promotion of lung adenocarcinoma cell migration and invasion.

To understand the effects of HNF1A-AS1 on the metastasis of lung adenocarcinoma *in vivo*, A549 cells stably transfected with sh-HNF1A-AS1 were injected into male nude mice. Seven weeks after injection, we counted the metastatic nodules on the surface of the lungs. HNF1A-AS1 knock-down led to a reduction in the number of metastatic nodules compared with the control group (Figure 5E, 5F). In addition, HE staining of lung sections also significantly decreased in the sh-HNF1A-AS1-transfected tumors (Figure 5G). Our *in vivo* results further confirmed the functional effects of *in vitro* studies involving HNF1A-AS1.

### DNMT1 binds to HNF1A-AS1

**Figure 6:**
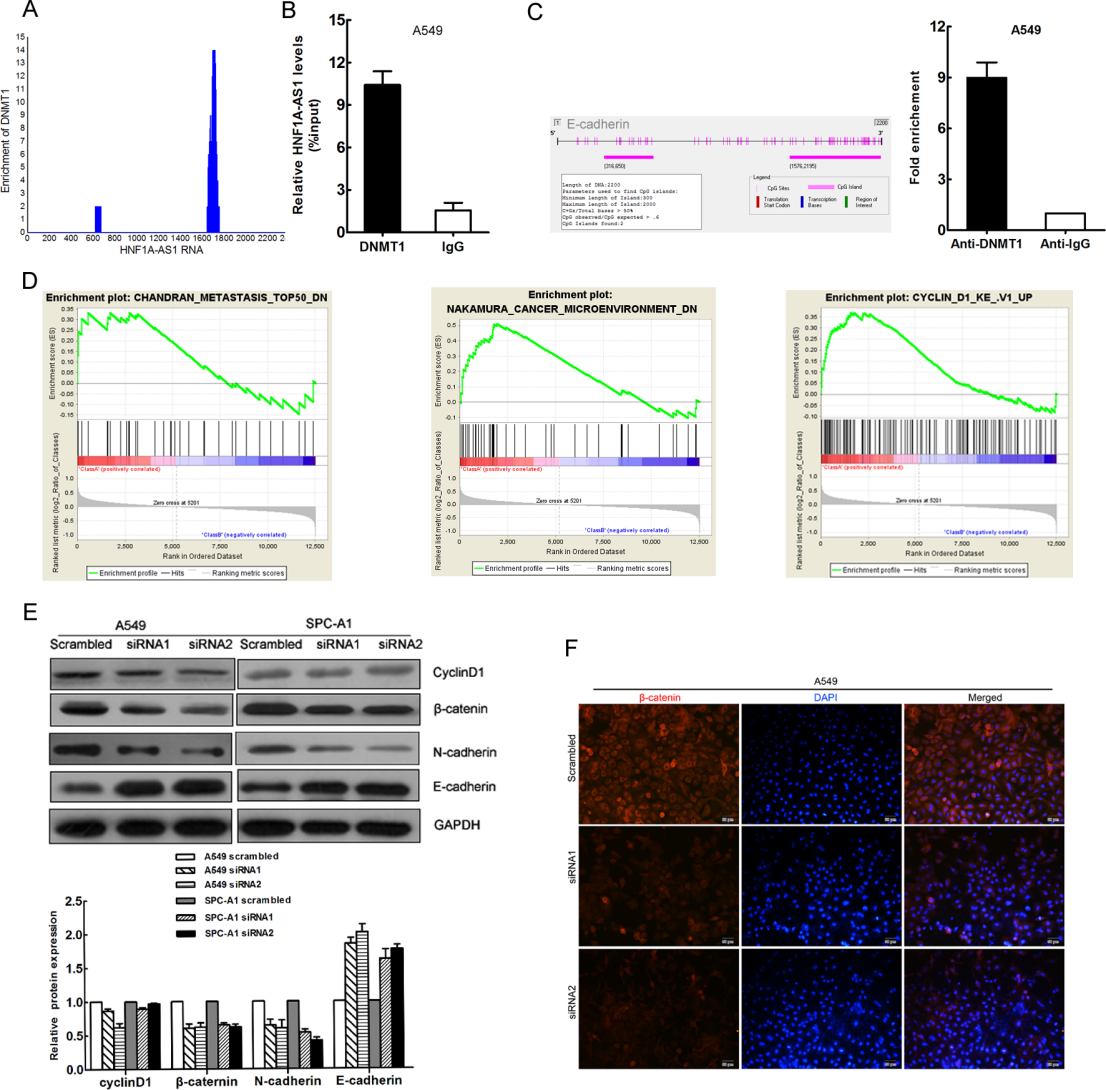
DNMT1 binds to HNF1A-AS1 HNF1A-AS1 influences EMT and Cyclin D1 in lung adenocarcinoma **(A)** Bioinformatics analysis showed HNF1A-AS1 was enriched in sequencing data of DNMT1 RIP-seq data (GSE32260) **(B)** RNA immunoprecipitation (RIP) experiments were performed in A549 using the anti-DNMT1 antibody and the coprecipitated RNA was subjected to qRT-PCR for HNF1A-AS1. RIP enrichment was determined as RNA associated with DNMT1 relative to the input control. **(C)** CpG islands were found in the E-cadherin promoter regions by Methyl Primer Express Software. ChIP–qPCR of DNMT1 binding to the E-cadherin promoter in A549 cells **(D)** Gene set enrichment analysis (GSEA) indicated that HNF1A-AS1 expression was associated with gene sets involved in metastasis, microenvironment and Cyclin D1. (data downloaded from http://www.ncbi.nlm.nih.gov/geo/query/acc.cgi?acc=GSE48240) **(E)** Analysis of cyclinD1, E-cadherin N-cadherin and β-catenin expression in A549 and SPC-A1 cells treated with siRNA HNF1A-AS1 by western blot. **(F)** Analysis of β-catenin expression in A549 cells treated with siRNA HNF1A-AS1 by immunofluorescence. *p < 0.05, **p < 0.01.

To further study the mechanism of HNF1A-AS1 regulation of lung adenocarcinoma cell proliferation and invasion, we employed bioinformatics analysis to validate the possible binding protein of the full-length HNF1A-AS1 transcript. We then searched the RIP-seq data (GSE32260) provided by Di Ruscio A at the GEO datasets. (http://www.ncbi.nlm.nih.gov/geo/query/acc.cgi?acc=GSE32260) The data correspond to RNA-binding signals from the DNMT1 protein measured in HL60 cell lines. The blast result showed that HNF1A-AS1 was enriched in the DNMT1 RIP-seq sequencing data (Figure 6A).

DNA methylation is a key epigenetic mechanism involved in transcriptional regulation that has been associated with a large number of human malignancies [20, 21]. DNA methyltransferase 1 (DNMT1), a member of the DNMT family, plays an essential role in DNA methylation, accounting for the majority of epigenetic maintenance and alteration [22]. DNMT1 has also been shown to be responsible for lung tumorigenesis [23, 24]. To determine whether DNMT1 physically associates with HNF1A-AS1, we performed RNA immunoprecipitation (RIP) from A549 cell lines with a specific anti-DNMT1 antibody. As shown in Figure 6B, we observed HNF1A-AS1 enrichment in DNMT1–RNA precipitates, demonstrating that a physical interaction occurs between HNF1A-AS1 and DNMT1.

### HNF1A-AS1 influences lung adenocarcinoma cell EMT

The gene set enrichment analysis was designed to show gene set differences between anti-HNF1A-AS1 siRNA- and scrambled siRNA-transfected OE33 cells whose data were downloaded from http://www.ncbi.nlm.nih.gov/geo/query/acc.cgi?acc=GSE48240. The results indicated that HNF1A-AS1 expression was associated with gene sets involved in metastasis, microenvironment, and cyclin D1 (Figure 6D).

First, we detected cyclin D1 protein expression by western blotting. We observed that cyclin D1 protein expression was only reduced in the siRNA2-treated A549 group, which is in agreement with the cell cycle results (Figure 6E). These data provide evidence that HNF1A-AS1 regulates cell cycle via cyclin D1.

The EMT is a major phenotypic marker of tumor metastasis and invasion in epithelial cancers, including lung adenocarcinoma [25]. E-cadherin is a particularly critical cell adhesion molecule that acts as an invasion suppressor [26]. UCSC Bioinformatics analysis has shown that CpG islands are present in the E-cadherin promoter regions (Figure 6C). Similar to previous studies which have demonstrated that E-cadherin expression is regulated by DNMT1 in cancer [27–29], our results of ChIP assays showed that DNMT1 could directly bind to E-cadherin promoter region (Figure 6C). Therefore, we conducted western blotting assays to detect the expression of E-cadherin and two other EMT-induced markers (N-cadherin, and β-catenin) in cells with down-regulated HNF1A-AS1 expression. Our findings indicated that decreased HNF1A-AS1 expression levels increased E-cadherin expression while decreasing N-cadherin and β-catenin expression (Figure 6E). Simultaneously, immunofluorescence analysis also revealed that reduced HNF1A-AS1 expression reduced β-catenin expression in A549 cells (Figure 6F). Western blotting analysis showed that EMT-related protein expression was increased in both cell lines treated with si-HNF1A-AS1 compared with the controls. Thus, E-cadherin may represent an important downstream effector of HNF1A-AS1, potentially mediated by DNMT1.

## DISCUSSION

It has become increasingly apparent that mammalian genomes encode thousands of lncRNAs that fulfill important functions in the regulation of gene expression. LncRNA dysregulation may be involved in epigenetics and participate in cancer growth and metastasis. Therefore, the identification and investigation of cancer-associated lncRNAs may provide new prognostic biomarkers or therapeutic strategies for cancer.

HNF1A-AS1 is a lncRNA (2455 bases in humans) identified by a next-generation sequencing analysis in esophageal adenocarcinoma. Yang et al. also reported that HNF1A-AS1 was up-regulated in esophageal adenocarcinoma and played a role in cell proliferation, cell cycle regulation, migration and invasion in esophageal adenocarcinoma [19]. However, the possible role and associated molecular mechanisms of HNF1A-AS1 in lung adenocarcinoma are yet to be clarified.

This report is the first direct investigation of the relationship between HNF1A-AS1 expression and lung adenocarcinoma. In this study, we found that the average level of HNF1A-AS1 in lung adenocarcinoma tissues was significantly higher than those in adjacent normal lung tissues. Specifically, HNF1A-AS1 expression was significantly higher in larger tumors and at later stages of tumor development. Moreover, the TCGA dataset also showed that HNF1A-AS1 expression could serve as a prognostic factor for lung cancer. In addition, HNF1A-AS1 knockdown significantly inhibited lung adenocarcinoma cell viability, G1–G0 phase arrest, migration, and invasion both *in vitro* and *in vivo*. Taken together, the observations of this study indicate that HNF1A-AS1 may serve as an oncogene and may play an important role in lung adenocarcinoma development and progression. Many other lncRNAs have also been implicated in the development of multiple tumors and identified as cancer biomarkers. For example, elevated expression of metastasis-associated lung adenocarcinoma transcript 1 (MALAT1) is associated with lung cancer progression and metastasis [30]. Another prominent example is Hox transcript antisense intergenic RNA (HOTAIR), pervasively overexpressed in most human cancers [31]. Thus, effective blocking of these lincRNAs in cancer could be a novel preventive and therapeutic strategy. Our animal model studies have further indicated that targeting HNF1A-AS1 could inhibit lung adenocarcinoma proliferation and metastasis. Recent advances in targeting natural antisense transcripts with siRNAs or antisense oligonucleotides may make this an exciting new approach to the treatment of lung adenocarcinoma.

Although HNF1A-AS1 has been suggested to act as an oncogene, the underlying mechanism by which HNF1A-AS1-mediated gene expression participates in tumorigenesis remains to be clarified. Yang and colleagues have provided evidence that the important cancer-related lncRNA H19 may represent a downstream effector of HNF1A-AS1 in esophageal adenocarcinoma. In this study, to explore the molecular mechanism by which HNF1A-AS1 contributes to the cell proliferation of lung adenocarcinoma cells, we investigated potential targets that could be responsible for cell cycle arrest and cell growth inhibition. The present experimental results confirmed that Cyclin D1 is a functional target of HNF1A-AS1 in lung adenocarcinoma. Cyclin D1 is one of the most important cell cycle regulatory proteins, playing pivotal roles in the development of a subset of human cancers including lung cancer [32, 33]. Cyclin D1 assembles with CDK4 and the CIP/KIP protein, which then enters the nucleus and phosphorylates tumor suppressor protein Rb, inducing progression from the G1 to the S phase [34]. Here, we found that Cyclin D1 was a downstream regulator involved in HNF1A-AS1-mediated growth arrest in lung adenocarcinoma. However, siRNA-mediated knockdown of HNF1A-AS1 neither promoted G1 arrest nor reduced cyclin D1 protein expression in SPC-A1. As lncRNAs have been known to be expressed in a spatial- or temporal-specific manner, cell-specific and developmental dynamic manner [35–37], their roles in regulating gene expression and biological processes differ in diverse cellular contexts [38]. To fully appreciate the functions of HNF1A-AS1, further studies are required to construct complete functional cell type-specific lncRNA expression maps in different steps of cancer progression.

While a previous study showed that increased expression of the lncRNA HNF1A-AS1 affects migration and invasion in esophageal adenocarcinogenesis, the underlying molecular mechanisms of this effect remain unknown. To explore the molecular mechanism by which HNF1A-AS1 contributes to the invasion and metastasis of lung adenocarcinoma, we explored potential target proteins involved in cell motility and invasion. An important program termed the epithelial–mesenchymal transition (EMT) has been shown to be crucial in promoting progression and metastasis in epithelium-derived carcinomas, including lung adenocarcinoma [3, 39–41]. The epithelial to mesenchymal transition is characterized by decreased E-cadherin and increased N-cadherin expression, contributing to increased tumor cell motility and invasive properties [42, 43]. Our findings indicated that inhibitory effects on cell migration and invasion were associated with EMT-related factors (E-cadherin, N-cadherin, β-catenin). Our findings demonstrated that the down-regulation of HNF1A-AS1 repressed NSCLC cell migration, invasion and metastasis through the EMT. This could provide a theoretical rationale for HNF1A-AS1 as a potential target for anti-metastatic therapies.

The function of lncRNAs usually relies on the proteins that they bind. For example, the lncRNA HOTTIP has been reported to bind directly to the WDR5 protein and target WDR5/MLL complexes, inducing histone H3 lysine 4 trimethylation and gene transcription [44]. HOTAIR and Xist. have also been shown to interact with the chromatin remodeling protein PRC2 complex to repress gene regulation [45]. In our study, we found that HNF1A-AS1 was mostly located in the cell nucleus, next revealed the abundant binding between HNF1A-AS1 and DNMT1 in A549 cells using RIP. DNMT1, the predominant mammalian DNA methyltransferase, maintains DNA methylation and gene silencing in human cancer cells [46, 47]. Di Ruscio et al. found that DNMT1 could interact with RNAs to block gene-specific DNA methylation [48]. As CpG islands were observed in the E-cadherin promoter regions], the Chip assays showed that DNMT1 could directly bind to E-cadherin promoter regions. Recently E-cadherin expression has also been proven to be regulated by DNMT1 in different cancer cells [28, 29]. Thus, HNF1A-AS1 may regulate E-cadherin through DNMT1. Since E-cadherin expression is known to be a critical step in the progression of well differentiated adenoma to invasive carcinoma [5], HNF1A-AS1 may mediate the binding of DNMT1 to E-cadherin which decreases the E-cadherin and induces EMT.

In summary, this study identifies HNF1A-AS1 as a novel potential oncogene in lung adenocarcinoma that acts by inducing the EMT process. HNF1A-AS1 may act as a prognostic factor in lung adenocarcinoma. We observed that the silencing of HNF1A-AS1 in lung adenocarcinoma cells dramatically blocked tumor growth and metastasis *in vivo* and *in vitro*, raising the possibility that HNF1A-AS1 could be a promising new therapeutic target for highly aggressive lung adenocarcinoma.

## MATERIALS AND METHODS

### Tissue collection

40 paired lung adenocarcinoma and corresponding adjacent non-tumor lung tissues were obtained from patients who underwent surgery in the Department of Thoracic Surgery, Jinling Hospital, Nanjing University School of Medicine, China, between 2013 and 2014. All the cases were diagnosed with lung adenocarcinoma based on histopathological evaluation. Clinicopathological characteristics, including tumor node metastasis staging, were available for all samples (Table 1). No patients had received local or systemic treatment before surgery. All collected tissue samples were immediately snap-frozen in liquid nitrogen and stored at −80°C until RNA extraction. The study protocol was approved by the Institutional Review Board of Nanjing University, China. Written informed consent was obtained from all participants.

### Cell lines and culture conditions

Five NSCLC cancer cell lines (A549, SPC-A1, H1650, H1703, SK-MES-1, and H520) and a normal human bronchial epithelial cell line (16HBE) were purchased from the Institute of Biochemistry and Cell Biology of the Chinese Academy of Sciences (Shanghai, China). Cells were maintained in RPMI 1640 (GIBCO-BRL; Invitrogen, Carlsbad, CA) or Dulbecco's modified Eagle's medium (DMEM; GIBCO-BRL; Invitrogen) supplemented with 10% fetal bovine serum (FBS) and antibiotics and cultured at 37°C in humidified air with 5% CO_2_.

### RNA extraction and quantitative reverse-transcriptase polymerase chain reaction (qRT-PCR) analyses

Total RNA was extracted from frozen tissues or cultured cells using TRIZOL reagent (Invitrogen, Carlsbad, CA). For qRT-PCR, the isolated RNA was reverse transcribed to cDNA using a Reverse Transcription Kit (Takara, Dalian, China). Real-time PCR analyses were conducted with Power SYBR Green (Takara, Dalian, China). The results were normalized to the expression of GAPDH. The primers were as follows: GAPDH sense 5′-GGGAGCCAAAAGGGTCAT-3′, reverse 5′-GAGTCCTTCCACGATACCAA-3′; HNF1-AS1 sense, 5′-TCAAGAAATGGTGGCTAT-3′, reverse 5′-GCTCTGAGACTGGCTGAA-3′. qRT-PCR and data collection were performed using an ABI 7500 instrument (Applied Biosystems, Foster City, CA). All qRT-PCR reactions were performed in duplicate.

### Subcellular fractionation location

The separation of nuclear and cytosolic fractions was carried out using the PARIS Kit (Life Technologies) according to the manufacturer's instructions. RNA was extracted from each fraction using TRIZOL reagent (Invitrogen, Carlsbad, CA).

### Transfection of cell lines

The siRNA (small interfering RNA) sequences were as follows: HNF1A-AS1 siRNA1 (5′-3′ CAC CUG CAU UCA AAC UCG GAC UGU U), HNF1A-AS1 siRNA2 (5′-3′ GGG UGA GCA GCU GUU UGC AAG ACU A). Synthetic sequence-scrambled siRNA from Invitrogen were used as negative controls. The HNF1A-AS1 and control siRNAs were transfected into A549 and SPC-A1 cells, which were cultured on six-well plates using Lipofectamine 2000 (Invitrogen, Shanghai, China) according to the manufacturer's protocol. The target sequence of shRNA-HNF1A-AS1 was as follows: CACCTGCATTCAAACTCGGACTGTT.

### Cell proliferation assays

A cell proliferation assay was performed using the MTT kit (Sigma, St. Louis, MO) according to the manufacturer's instruction. Cells were plated into 6-well plates and cultured in media containing 10% FBS, which was replaced every 4 days. After 14 days, colonies were fixed with methanol and stained with 0.1% crystal violet (Sigma, St. Louis, MO). Visible colonies were then manually counted. For each treatment group, wells were counted in triplicate.

### Flow-cytometric analysis of apoptosis and the cell cycle

A549 and SPC-A1 cells transiently transfected with siRNA were harvested 48 h after transfection by trypsinization. Following the double staining with FITC-Annexin V and Propidium Iodide (PI), the cells were analyzed by flow cytometry (FACScan^®^; BD Biosciences, San Jose, CA) equipped with CellQuest software (BD Biosciences). Cells were categorized as early apoptotic cells, late apoptotic cells, dead cells, or viable cells. The ratio of early apoptotic cells to late apoptotic cells was compared to that for controls from each experiment. For the cell cycle analysis, the cells were stained with PI using the CycleTEST™ PLUS DNA reagent kit (BD Biosciences) according to the manufacturer's protocol. The ratio of cells in the G0/G1, S, and G2/M phases were counted and compared. All of the samples were assayed in triplicate.

### Cell migration and invasion assays

At 24 h after transfection, cells in serum-free media were seeded into the upper chamber for migration assays (8 μm pore size, Millipore) and invasion assays with Matrigel (Sigma-Aldrich, USA). The lower chambers were filled with media containing 10% FBS. After several hours of incubation at 37°C, the cells that had migrated or invaded through the membrane were fixed in paraformaldehyde and stained with 0.1% crystal violet (Sigma). The cells on the lower surface were photographed and five random fields were counted. Three independent experiments were performed.

### Western blotting

Cells were lysed using a lysis buffer containing the mammalian protein extraction reagent RIPA (Beyotime China), a protease inhibitor cocktail (Roche, Basel, Switzerland) and PMSF (Roche). The protein concentration was detected using a Bio-Rad protein assay kit. Samples containing 40 μg of protein from two different cell lines were electrophoresed on a 10% SDS-polyacrylamide gel (SDS-PAGE) and then transferred onto 0.22 μm nitrocellulose membranes (Sigma-Aldrich) and incubated with specific antibodies. The ECL chromogenic substrate was used to detect specific bands. Protein expression was quantified using densitometry (Quantity One Software; Bio-Rad, Hercules, CA), with GAPDH used as a control. Additionally, antibodies (1:1000 dilution) against E-cadherin, N-cadherin and β-catenin were purchased from Cell Signaling Technology (MA, USA). Anti-cyclin D1 was obtained from Bioss (Beijing, China).

### Immunofluorescence

Cells were fixed in 4% paraformaldehyde according to the manufacturer's instructions. Rabbit anti-β-catenin antibodies (1:100; CST) were used as primary antibodies, with TRITC-labeled anti-Rabbit IgG (1:200; Sigma) used as a secondary antibody. Sections mounted onto slides using Gel Mount Aqueous Mounting Medium (G0918, Sigma) were imaged with an Olympus IX71 microscope (Olympus Optical, Tokyo, Japan).

### RNA binding protein immunoprecipitation (RIP) assay

RIP experiments were performed using the Magna RIP™ RNA-Binding Protein Immunoprecipitation Kit (Millipore, Billerica, MA, USA) according to the manufacturer's protocol. Antibodies used in the DNMT1 RIP assays were obtained from Abcam. A549 cells were harvested from 15 cm plates and lysed in complete RIP lysis buffer. After centrifuging at 14,000 g for 10 min at 4°C, an aliquot (10%) of supernatant was removed and tested. A 100 μl sample of whole cell extract was removed and incubated with RIP buffer containing magnetic beads conjugated with human anti-DNMT1, negative control normal mouse IgG (Millipore) and positive control anti-SNRNP70. Samples were incubated with Proteinase K buffer with shaking to digest the protein, and then immunoprecipitated RNA was purified. The RNA concentration and quality were measured using a NanoDrop (Thermo Scientific). Purified RNA was analyzed by qRT-PCR to determinate the presence of the binding targets using gene specific primers.

### Chromatin immunoprecipitation

A549 cells were treated with formaldehyde and incubated for 10 minutes, followed by sonication to achieve the majority of DNA fragments with 200 to 300 bp. The fragmented chromatin were immunoprecipitated with DNMT1 antibody (Abcam) or IgG as control. Precipitated chromatin DNA was recovered and measured by qPCR.

### Bioinformatics methods

The UCSC Cancer Genomics Browser can be accessed at https://genome-cancer.ucsc.edu/proj/site/hgHeatmap/. Genomic and clinical data can be visualized according to the specific gene using the genes viewing mode [49].

The RIP-sequence data (GSE32260) predicting the possibility of HNF1A-AS1 interactions with DNMT1 were obtained from http://www.ncbi.nlm.nih.gov/geo/query/acc.cgi?acc=GSE32260.

Microarray data (GSE48240) for HNF1A-AS1 knockdown in OE33 cells was downloaded (http://www.ncbi.nlm.nih.gov/geo/query/acc.cgi?acc=GSE48240) and gene set enrichment analysis (GSEA) was performed on various gene signatures by GSEA v2.0. CpG islands in the E-cadherin promoter regions were analyzed by Methyl Primer Express v1.0.

### Tumor formation assay in a nude mouse model

Male athymic BALB/c nude mice (6 weeks old) were used for the tumor formation assay. The animal care and experimental protocols were approved by the Model Animal Research Center of Jingling Hospital and carried out in strict accordance with Institutional Animal Care and Use guidelines. A549 cells stably transfected with HNF1A-AS1 shRNA (GeneChem, Shanghai, China) or empty vector were cultured in six well plates for 48 h. Then, the cells were washed with phosphate-buffered saline and resuspended at 1 × 10^8^ cells/ml. A total of 100 μl of suspended cells was subcutaneously injected into the right side of the posterior flank of each mouse. Tumor volumes and weights were measured beginning from day 3 after the tumor cell injection. At 14 days post injection, the subcutaneous growth of each tumor was measured. Tumor volumes were calculated as length × width^2^ × 0.5. All surgeries were performed under sodium pentobarbital anesthesia, and every effort was made to minimize animal suffering [50].

### Tail vein injections into athymic mice

A549 cells stably transfected with HNF1A-AS1shRNA or the empty vector were harvested from 6-well plates and collected at 2 × 10^7^ cells/ml. Suspended cells (100 μl) were injected into the tail veins of 14 mice (4 weeks old), which were sacrificed 7 weeks after injection. The lungs were removed and visible tumors on the lung surface were counted and used for further analysis.

### Statistical analysis

Two-tailed Student's *t*-tests (two tailed) and one-way ANOVAs were used to analyze data with SPSS 22.0 software (IBM, Chicago, IL, USA). *P*-values of less than 0.05 were considered to be statistically significant.
